# The Impact of Social Participation on Frailty among Older Adults: The Mediating Role of Loneliness and Sleep Quality

**DOI:** 10.3390/healthcare12202085

**Published:** 2024-10-18

**Authors:** Yanting Wang, Feiyang Zheng, Xinping Zhang

**Affiliations:** School of Medicine and Health Management, Huazhong University of Science and Technology, Wuhan 430000, China; m202275717@hust.edu.cn (Y.W.); feiyangzheng@hust.edu.cn (F.Z.)

**Keywords:** social participation, frailty, loneliness, sleep quality, mediating role, older adults

## Abstract

Background: Frailty has become a common health issue among older adults, imposing a burden on both society and individuals. The relationship between social participation and frailty has received widespread attention, but the mechanism remains to be explored. The aim of this study is to explore the impact of social participation on frailty among older adults and to analyze the mediating role of loneliness and sleep quality, providing suggestions to alleviate frailty. Methods: Data related to social participation, loneliness, sleep quality, and frailty from 7779 older adults were collected from the Chinese Longitudinal Healthy Longevity Survey (CLHLS 2018). The chain mediation model was conducted to explore the relationship between variables, and the Bootstrap method was used to examine the path coefficients. Results: Social participation negatively affected frailty (β = −0.00391049, 95% CI = [−0.042296, −0.035465]); the indirect effect of social participation on frailty mediated by loneliness was −0.0019505 (95% CI = [−0.002551, −0.001371]); the indirect effect of social participation on frailty mediated by sleep quality was −0.0011104 (95%CI = [−0.001692, −0.000557]); the effect mediated by both loneliness and sleep quality was −0.0004263 (95% CI = [−0.000593, −0.000304]). Conclusions: Social participation negatively affected frailty. Loneliness and sleep quality not only mediated independently, but also played a chain mediating role. This suggested that encouraging older adults to engage in more social participation, reducing loneliness, and improving sleep quality are feasible measures to improve frailty.

## 1. Introduction

Frailty refers to the decline in functions of multiple body systems that occurs with age [1], which can result in the risk of a series of negative health outcomes such as death and increased medical costs, placing an increasing burden on society and individuals’ health and well-being [2]. Frailty is increasingly becoming common among older adults. According to surveys, the prevalence of frailty is 8.7% in Japan [3] and 10% in China [2] among older adults. With global population aging accelerating, it is crucial and urgent to search for measures to prevent and alleviate the development of frailty.

China is a developing country with the largest aging population, and in this context, frailty is a more serious and urgent public health issue [4]. Social participation is a key component for healthy aging and an important factor to be considered in future healthcare services [5]. There is growing interest in the relationship between social participation and frailty. Previous scholars have explored the relationship through horizontal and vertical research [6,7,8,9], and the impact of different types and frequencies of social participation on frailty [10,11] and the impact of different patterns of social participation on frailty have been studied [12]. A few scholars also have conducted research on the influencing mechanism between social factors and frailty, such as the mediating effect of loneliness on social isolation and frailty [13]. However, it is important to note that the mechanisms by which social participation affects frailty have not been fully elucidated.

Loneliness refers to the unpleasant psychological experience that occurs when there is a lack of quantity or quality in one’s social network [14]. In the context of the traditional Chinese family structure and collective culture, family members are the main way of receiving support [15]. However, with the development of the economy and the acceleration of urbanization, empty-nester and floating older adults are becoming increasingly common in China, which is making loneliness a common phenomenon among older adults [16]. More social participation has become an important way to compensate for the lack of support. Previous studies have found that individuals with higher levels of social participation are more likely to experience lower levels of loneliness [8,15,17,18]. Meanwhile, some empirical studies have shown that loneliness is an important factor in the onset and development of frailty [8,19,20]. These studies suggest that loneliness may play a mediating role in the impact of social participation on frailty.

Poor sleep quality has become one of the most common problems among older adults [21]. Scholars have begun to take an interest in the critical role of social factors on sleep quality. As it has been documented, participating in interest groups, community organizations, and high-frequency exercise groups are all associated with better sleep quality [22]; a high level of social capital leads to better sleep quality [23]. Sleep quality is also closely related to frailty. Empirical studies have shown that poorer sleep quality leads to higher levels of frailty in older adults [24,25]. Given the association between sleep quality and social participation and frailty, we assumed that sleep quality may be a mediating variable.

In addition, loneliness affects sleep quality. It has been shown that loneliness can lead to poor sleep quality [26]. Related reviews also indicated that poor sleep quality is more likely to be experienced by older adults who are lonely than those who do not feel lonely [27]. Another review found that loneliness and social isolation can both influence sleep quality, and loneliness has a greater impact [28]. In addition, according to the senescent sleep model which elucidates the factors that affect sleep complaints in older adults, loneliness can have adverse effects on the sleep–wake cycle and make sleep complaints permanent [29]. Therefore, loneliness and sleep quality may play a chain mediating role.

In summary, the association between social participation and frailty has garnered considerable attention. As mentioned above, there is a correlation between social participation, loneliness, sleep quality, and frailty. Nonetheless, there is a lack of research that examines the mediating role of sleep quality and loneliness in the impact of social participation on frailty.

Therefore, this study aims (1) to examine whether social participation has an impact on frailty; (2) to explore whether loneliness and sleep quality play mediating roles in the impact of social participation on frailty; (3) to explore whether loneliness and sleep quality play a chain mediating role in the impact of social participation on frailty.

## 2. Methods

### 2.1. Data and Sample

The data of this study were obtained from the Chinese Longitudinal Healthy Longevity Survey (CLHLS 2018) database, jointly performed by the Center for Healthy Aging and Development Studies at Peking University and Duke University, covering 23 provinces (municipalities and autonomous regions) in China. Considering the needs of our research, we included participants who were (1) age ≥ 60 years old and were (2) without missing values on the key variables such as social participation, loneliness, sleep quality, frailty, and sociodemographic information; thus, a total of 7779 individuals were included.

### 2.2. Measures

#### 2.2.1. Social Participation

The independent variable for this study was social participation, which includes tai chi, square dancing, visiting and interacting with friends, other outdoor activities, gardening, reading newspapers/books, raising domestic animals/pets, playing cards/mah-jong, watching TV or listening to the radio, and social activities. Answers ranged from 0 to 4 (0 = never, 4 = almost every day). The level of social participation was obtained by summing up the scores of each item, with higher scores indicating higher levels of social participation.

#### 2.2.2. Frailty

The dependent variable was frailty. In this study, the method of measuring frailty was to calculate the frailty index (FI), which is defined as the proportion of existing deficits in relation to all included deficits [30]. Based on previous studies [1,30], 35 health deficits were used to construct the FI in our study, including self-rated health, psychological characteristics, activities of daily living (ADL), instrumental activities of daily living (IADL), hearing or vision impairment, cognitive functioning, and chronic diseases. The Cronbach’s alpha coefficient of the FI was 0.711, which indicates that the reliability was acceptable. The higher the FI, the higher the degree of frailty.

#### 2.2.3. Mediators

Loneliness was the first mediator variable taken into account in this study. It was measured by a single question, “Do you often feel lonely?”, with a total score range of 1–5 points. Higher scores represented higher loneliness. This single-item measure has been demonstrated to correlate highly with multi-item loneliness scales and has been widely used in previous studies [15,18].

Sleep quality was another mediator variable. It was measured by using the question, “How is your sleep quality now?”, with a total score range of 1–5 points. The higher the score, the greater the sleep quality.

#### 2.2.4. Control Variables

The control variables in this study included age (in years), gender (male = 1, female = 0), marital status (without spouse = 0, have spouse = 1), education (no formal education = 1, 1 to 6 = 2, 7 or more = 3), and residence (urban = 1, rural = 2).

### 2.3. Statistical Analysis

The sample size was calculated for linear multiple regression with a medium effect size set to 0.15, significance level (alpha) set to 0.05, and power (beta) set to 0.8 by using the statistical software R version 4.4.1. The sample required for this analysis was 107, and a total sample of 7779 individuals was included in this study. We used STATA version 17.0 software to perform the following statistical analysis. Firstly, we conducted descriptive analysis on the variables used in this study, with age, social participation, frailty, loneliness, and sleep quality presented as mean and standard deviation, and gender, marital status, education level, and place of residence presented as frequency and percentage. Secondly, the correlation between social participation, loneliness, sleep quality and frailty were investigated by using Pearson correlation analysis. Finally, a chain mediation model analysis was used to conducted to investigate potential mechanisms between social participation and frailty, disaggregating the direct and indirect pathways of this association. The total and indirect effects were tested using bootstrapping with 1000 iterations. A *p*-value of less than 0.05 was considered statistically significant.

## 3. Results

### 3.1. Descriptive Analysis

In this study, the mean level of social participation, frailty, loneliness, and sleep quality was 9.36 ± 6.35, 0.15 ± 0.08, 1.91 ± 0.97, and 3.55 ± 0.98, respectively. See Table 1 for details.

### 3.2. Correlation Analysis

As is shown in Table 2, there was a significant correlation (*p* < 0.01) between social participation, loneliness, sleep quality and frailty. Social participation was negatively correlated with frailty (r = −0.371, *p* < 0.01) and loneliness (r = −0.141, *p* < 0.01) and positively correlated with sleep quality (r = 0.073, *p* < 0.01). Loneliness was positively correlated with frailty (r = 0.236, *p* < 0.01) and negatively correlated with sleep quality (r = −0.216, *p* < 0.01). Sleep quality was negatively correlated with frailty (r = −0.202, *p* < 0.01).

### 3.3. Mediation Analysis

**Figure 1:**
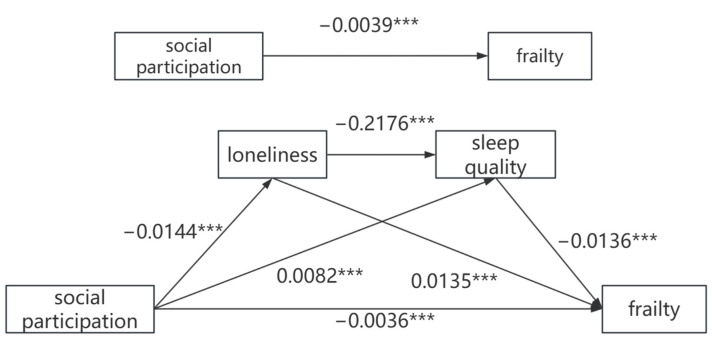
Chain mediation model of social participation and frailty through loneliness and sleep quality. The rectangular boxes represent observed variables, single headed arrows represent paths between variables, and *** represents the *p*-value of the path coefficient is less than 0.01.

The analysis of the chained mediation model consisted of four multiple linear regressions, with control variables included. Figure 1 and Table 3 show the results of the chained mediation effects analysis. Social participation was a significant predictor of frailty in older adults (β = −0.0039, *p* < 0.01) in the total effects model. This relationship still held when loneliness and sleep quality were also taken into account (β = −0.0036, *p* < 0.01). In addition, social participation had a significant effect on loneliness (β = −0.0144, *p* < 0.01) and sleep quality (β = 0.0082, *p* < 0.01). Loneliness and sleep quality were significant factors for frailty, with corresponding coefficients of 0.0135 (95%CI = [0.0118,0.0153], *p* < 0.01) and −0.0136, (95%CI = [−0.0153, −0.0119], *p* < 0.01), respectively.

We verified the total, direct, and indirect effects using the bootstrap method. Specifically, the indirect effect of social engagement mediated by loneliness on frailty in older adults was −0.0019505 (95% CI = [−0.002551, −0.001371]); the indirect effect of social engagement mediated by sleep quality on frailty in older adults was −0.0011104 (95%CI = [−0.001692, −0.000557]); the chain-mediated effect co-mediated by loneliness and sleep quality was −0.0004263 (95% CI = [−0.000593, −0.000304]); the total indirect, direct, and total effects of social participation on frailty were −0.0034872 (95% CI= [−0.004424, −0.002661]), −0.0356177 (95% CI = [−0.038594, −0.032189]), −0.0391049 (95% CI = [−0.042296, −0.035465]), respectively. See Table 4.

## 4. Discussion

This is the first study to examine the mechanisms by which social participation affects frailty from the perspective of loneliness and sleep quality, as far as we know. Our findings suggested that social participation reduces levels of frailty and that loneliness and sleep quality play mediating roles among older adults.

### 4.1. The Negative Impact of Social Participation on the Frailty

Our study showed that a higher level of social participation leads to a lower level of frailty (effect = −0.0391049, 95%CI = [−0.042296, −0.035465]), which is consistent with other studies. Previous studies have shown that older adults who interact with friends, participate in hobby groups, join sports groups, and volunteer have a lower risk of frailty [6,9]. This may be due to the fact that social participation can lower the risk of depression [8]; can promote the more effective use of brain networks, reducing the risk of dementia [31]; and is associated with a lower incidence of disability, the slower progression of disability, and improved physical functioning [5,13], which are all predictive of lower frailty. In addition, social participation provides older people with a sense of companionship and promotes their social integration, which can have a positive impact on their health [9]. Although some studies suggested that social participation may have potential negative impacts, such as individual exposure to infectious diseases through interactions between people [32], it needs to be recognized that maintaining social participation is a key part of successful aging. In order to promote the long-term health of older adults, more attention should be paid to how to promote their social participation. Our research findings indicated that social participation is a feasible and effective measure to prevent and improve frailty, suggesting that healthcare providers can encourage older adults to engage in social participation more.

### 4.2. The Mediating Role of Loneliness

Our findings suggested that loneliness mediated the effects of social participation on frailty (effect = −0.0019505, 95% CI = [−0.002551, −0.001371]). This study showed that higher levels of social participation can lead to lower levels of loneliness and lower levels of frailty. Although this relationship has rarely been examined, previous research supported the results. For example, Teh et al. pointed out that in comparison, older adults who regularly watched television or listened to the radio had lower levels of loneliness due to the fact that watching television and radio can provide pleasure, and that in China, older adults usually watch television with family members and discuss television programs, which may reduce loneliness among older adults [18]. High-frequency social interaction activities may help older adults to build their social networks, increase contact with others, and motivate mutual support, thereby reducing loneliness, which may improve mental health or prevent depression [10]. Research suggested that loneliness is related to cognitive decline and difficulties in daily life activities [33]. Loneliness also provides a physiological basis for age-related frailty syndromes through biological processes such as the inflammatory system [34]. Our study provided empirical evidence that social participation influences frailty through loneliness, which suggests health intervention strategies should pay more attention to lonely older adults.

### 4.3. The Mediating Role of Sleep Quality

Sleep quality mediated the association between social participation and frailty (effect = −0.0011104, 95%CI = [−0.001692, −0.000557]). The empirical results showed that higher levels of social participation were linked to better sleep quality and lower levels of frailty. Previous research has noted that emotional support, resources, and health-promoting behaviors obtained from social participation can support good sleep quality [35] and that socio-intellectual activities (e.g., playing chess and cards) affect the brain’s ability to alter neural connections which regulate the body’s need for sleep, affecting an individual’s sleep [36]. In addition, as reported by some studies, poor sleep quality may lead to daytime dysfunction and muscle weakness [25] and depression [37] in older adults. And studies have also shown that poor sleep quality can lead to a series of problems related to chronic diseases, such as changes in autonomic activity, appetite regulation, and inflammation [38]. All of these adverse outcomes lead to an increase in the degree of frailty. This study revealed, for the first time, the mediating role of sleep quality between social participation and frailty in older adults, which indicates that we can not only alleviate the development of frailty by encouraging social participation among older adults, but also improve their sleep quality to improve frailty. Additionally, this study also found that sleep quality was a weaker mediator in the relationship between social participation and frailty. But this finding was not investigated in previous research, and we cannot provide a reliable and strong evidence to explain it.

### 4.4. The Chain Mediating Role of Loneliness and Sleep Quality

This study also explored the chain mediating effects of loneliness and sleep quality (effect = −0.0004263, 95% CI = [−0.000593, −0.000304]). This finding indicated that older adults with higher social participation report lower loneliness, which leads to better sleep quality, and as a result, improving frailty. Previous studies have indicated that social participation can reduce the risk of loneliness in older adults, which decreases the likelihood of poor sleep quality [5,15]. A previous path analysis in the Chinese population reported that loneliness and sleep quality play a chain mediated role in the relationship between activities of daily living (ADLs) and psychological distress in older adults [26]. Kim et al. also showed that social participation can improve insomnia symptoms by reducing loneliness [36]. The association between loneliness and sleep quality can also be explained by physiological mechanisms. It was reported that loneliness causes individuals to secrete chemicals that are secreted during wakefulness, which negatively affects falling asleep and is detrimental to quality sleep [39]. These previous studies provided evidence to support the chain mediating role of loneliness and sleep quality in the impact of social participation on frailty. Our study revealed this mechanism for the first time and is the first to provide quantitative evidence, supplementing the relevant literature and providing a deeper understanding of the mechanisms through which frailty can be influenced. And this finding suggested that it is necessary to focus on both loneliness and sleep quality to prevent and improve frailty.

The strength of this study is that it provided new knowledge to the current literature by examining, for the first time, the mediating role of loneliness and sleep quality on the link between social participation and frailty. At the same time, this study has some limitations. First, as this is a cross-sectional study, we cannot infer causality. Future longitudinal studies could be conducted to explore the directionality. Second, given that loneliness is a multidimensional and complex concept, the measurement of using a single-item scale may need to be improved. Future research may consider using standardized scales to measure loneliness.

## 5. Conclusions

This study aims to explore how social participation affects frailty in older adults and to investigate the mediating role of loneliness and sleep quality. This study confirms that higher social participation is associated with lower frailty. In addition, we found, for the first time, that loneliness and sleep quality mediate the relationship between social participation and frailty, independently and sequentially, thus gaining a deeper understanding of the mechanisms by which social participation affects frailty. Specifically, high-level social participation can reduce loneliness among older adults, thereby reducing their frailty. High-level social participation can reduce frailty in older adults by improving their sleep quality. High-level social participation can reduce frailty through a chain mediating effect of reducing loneliness and improving sleep quality.

Our results have important practical significance in providing guidance and insights for improving frailty. According to these results, the onset and development of frailty can be prevented and alleviated from the following aspects. Local governments and community organizations should encourage older adults, especially those with lower levels of social participation, to enhance participation in diverse social activities. Related clinics can provide regular psychological support and counseling services for older adults, such as organizing mental health lectures and support groups, to reduce their loneliness and help them better cope with it. Clinics can provide sleep health education for older adults, offering guidance on good sleep habits and environment to help improve their sleep quality. At the same time, clinics can provide screening and evaluation services for sleep quality to help detect potential health problems as early as possible, providing corresponding guidance and suggestions, and helping them improve their sleep.

## Figures and Tables

**Table 1:** The descriptive analysis of variables.

Variable	Mean (SD)/Frequency (%)
Social participation	9.36 (6.35)
Frailty	0.15 (0.08)
Loneliness	1.91 (0.97)
Sleep quality	3.55 (0.98)
Age (years)	83.14 (11.37)
Gender	
Male	3629 (46.65%)
Female	4150 (53.35%)
Marital status	
Without spouse	4096 (52.65%)
Has spouse	3683 (47.35%)
Education	
No formal education	3382 (43.48%)
1 to 6	2725 (35.03%)
7 or more	1672 (21.49%)
Residence	
Urban	1932 (24.84%)
Rural	5847 (75.16%)

**Table 2:** Correlations among study variables.

	Social Participation	Loneliness	Sleep Quality	Frailty
Social participation	1			
Loneliness	−0.141 ***	1		
Sleep quality	0.073 ***	−0.216 ***	1	
Frailty	−0.371 ***	0.236 ***	−0.202 ***	1

Note: *p*-values: *** *p* < 0.01.

**Table 3:** Multiple linear regression results.

	Frailty	Loneliness	Sleep Quality	Frailty
Social participation	−0.0039 *** [−0.0042, −0.0036]	−0.0144 *** [−0.0183, −0.0105]	0.0082 *** [0.0042, 0.0121]	−0.0036 *** [−0.0039, −0.0033]
Loneliness			−0.2176 *** [−0.2401, −0.1950]	0.0135 *** [0.0118, 0.0153]
Sleep quality				−0.0136 *** [−0.0153, −0.0119]
Age	0.0017 *** [0.0015, 0.0019]	−0.0079 *** [−0.0103, −0.0055]	0.0037 *** [0.0013, 0.0062]	0.0019 *** [0.0017, 0.0021]
Gender	0.0016 [−0.0022, 0.0054]	−0.0801 *** [−0.1268, −0.0334]	−0.2266 *** [−0.2740, −0.1792]	−0.0002 [−0.0038, 0.0035]
Marital status	0.0008 [−0.0034, 0.0050]	−0.4816 *** [−0.5337, −0.4295]	−0.0812 *** [−0.1350, −0.0273]	0.0077 *** [0.0035, 0.0118]
Education	0.0007 [−0.0021, 0.0035]	−0.0635 *** [−0.0981, −0.0289]	0.0071 [−0.0279, 0.0422]	0.0019 [−0.0008, 0.0046]
Residence	−0.0260 *** [−0.0303, −0.0218]	−0.0173 [−0.0701, 0.0355]	−0.0256 [−0.0791, 0.0280]	−0.0261 *** [−0.0302, −0.0220]
Constant	0.0886 *** [0.0637, 0.1135]	3.1984 *** [2.8901, 3.5068]	3.9983 *** [3.6778, 4.3188]	0.0902 *** [0.0646, 0.1157]
Observations	7779	7779	7779	7779
R square	0.2012	0.0646	0.0637	0.2579

Note: *p*-values: *** *p* < 0.01.

**Table 4:** Bootstrap test results for multiple intermediary models.

Path	Observed Coefficient	Bootstrap Standard Error	95%CI	
			Lower	Upper
Indirect effect via loneliness	−0.0019505	0.00003006	−0.002551	−0.001371
Indirect effect via sleep quality	−0.0011104	0.00002943	−0.001692	−0.000557
Indirect effect via loneliness and sleep quality	−0.0004263	7.115 × 10^−6^	−0.000593	−0.000304
Total indirect effect	−0.0034872	0.00004481	−0.004424	−0.002661
Direct effect	−0.0356177	0.00016162	−0.038594	−0.032189
Total effect	−0.0391049	0.00016922	−0.042296	−0.035465

## Data Availability

Publicly available datasets were analyzed in this study. These data can be found here: http://opendata.pku.edu.cn (accessed on 1 August 2024).
